# Differences in Learning Strategies, Goal Orientations, and Self-Concept between Overachieving, Normal-Achieving, and Underachieving Secondary Students

**DOI:** 10.3389/fpsyg.2016.01438

**Published:** 2016-09-27

**Authors:** Juan L. Castejón, Raquel Gilar, Alejandro Veas, Pablo Miñano

**Affiliations:** Department of Developmental Psychology and Didactic, University of AlicanteAlicante, Spain

**Keywords:** underachievement, overachievement, identification, individual variables, differential characteristics

## Abstract

The aims of this work were to identify and establish differential characteristics in learning strategies, goal orientations, and self-concept between overachieving, normal-achieving and underachieving secondary students. A total of 1400 Spanish first and second year high school students from the South-East geographical area participated in this study. Three groups of students were established: a group with underachieving students, a group with a normal level of achievement, and a third group with overachieving students. The students were assigned to each group depending on the residual punctuations obtained from a multiple regression analysis in which the punctuation of an IQ test was the predictor and a measure composed of the school grades of nine subjects was the criteria. The results of one-way ANOVA and the Games-Howell *post-hoc* test showed that underachieving students had significantly lower punctuations in all of the measures of learning strategies and learning goals, as well as all of the academic self-concept, personal self-concept, parental relationship, honesty, and personal stability factors. In contrast, overachieving students had higher punctuations than underachieving students in the same variables and higher punctuations than normal-achieving students in most of the variables in which significant differences were detected. These results have clear educational implications.

## Introduction

No definition for underachievement has been accepted by the entire scientific community (McCoach and Siegle, 2011). In the scientific literature, there is general agreement that underachievement is a discrepancy between what can be expected and what is actually achieved (McCoach and Siegle, 2003b, 2011). However, there has been a diversification of assumptions, as regarding studies related to the operationalization of the concept (Ziegler et al., 2012), the possible inclusion of students with learning disabilities into the underachievement framework (Fletcher et al., 2005) and the analysis of underachieving students with emotional and behavior disorders (Lane et al., 2002).

Given the multiple and specific conceptions of the construct, underachievement is a multidimensional construct that involves several variables. Analyses of these variables have been focused on underachieving gifted students (Chan, 1999; Ziegler and Stoeger, 2003; Dixon et al., 2006; Obergriesser and Stoeger, 2015), especially in the United States (Reis and McCoach, 2000; McCoach and Siegle, 2003a; Figg et al., 2012; Reis and Greene, 2014); however, the authors of the present work, in agreement with Dittrich (2014), support the assertion that underachievement is not reserved to gifted students but to all students situated at various intelligence levels, as they are also influenced by personal factors, family-related factors, and school-related factors. The treatment of these factors through educational interventions could lead to a better self-concept and academic achievement (Rodríguez et al., 2014; Álvarez et al., 2015; Veas et al., 2015).

The identification of underachieving students emerges as the principal basis to define its differential characteristics and to reverse the intervention. From a methodological perspective, the traditional statistical methods include the *absolute split method*, the *simple difference method* and the *regression method* (Lau and Chan, 2001; McCoach and Siegle, 2011).

When using the absolute split method, the researcher uses an arbitrary limit for the highest academic performance (e.g., top 5%) and the bottom academic performance (e.g., bottom 5%) after the conversion of punctuations to standard scores. This method has been used specifically in studies on gifted underachieving students (Peterson and Colangelo, 1996; Vlahovic-Stetic et al., 1999).

The simple difference score method is based on the discrepancy between the standardized performance score and the standardized ability score. When this difference is greater than an arbitrary limit (normally 1 standard deviation), a student could be regarded as underachieving (*d* < −1) or overachieving (*d* > 1). According to Lau and Chan (2001), this method is more appropriate than the absolute method for the identification of underachievement at all levels of ability. However, some researchers (McCall et al., 1992) have noted that this method may overestimate the number of underachieving students of above-average ability and underestimate the number of underachieving students of below-average ability.

The regression method is one of the most common methods to quantify the discrepancy between ability or expected achievement and actual achievement (Lau and Chan, 2001; McCoach and Siegle, 2011). This method is based on the deviation of the students' score from the regression line of the achievement measure based on the ability measure. Students are considered to be underachieving if this deviation is negative and greater than one standard error of the estimate. This method appears to have better reliability than the method of simple difference scores; however, it also generates a constant proportion of underachieving students (Plewis, 1991; Ziegler et al., 2012). Using this approach, over 15% of students would be identified as underachievers. However, Lau and Chan (2001) found a high degree of congruence among the three statistical methods (absolute split, difference score, and regression-based methods).

Others researchers (Anastasi, 1976; Fletcher et al., 2005; Ziegler et al., 2012) also highlighted ongoing issues of reliability and validity. They argued that underachievement is a latent variable and hence imperfectly measured using test instruments such as tests of intellectual ability and achievement. The assessment of underachievement must take into account problems of test reliability and measurement errors arising out of questions such as assumed normality. Furthermore, these measurement errors are compounded when two or more tests are used concurrently in assessing underachievement (Phillipson, 2008).

Adherence to the requirements of fundamental measurement is needed when using instruments that address underachievement. These measurement requirements include the need for unidimensionality of the measurement instrument and units of measurement that correspond to an interval scale.

When underachievement is defined as the discrepancy between expected achievement and actual achievement, the measurement of academic achievement must also meet the above requirements. There are two primary methods to assess achievement: standardized achievement tests and classroom or academic grades. External evaluations of the Autonomous Communities in Spain evaluate the skills mandated by existing legislation, particularly Constitutional Law 8/2013 on Improving Educational Quality (LOMCE, 2013^1^); however, schools continue to evaluate these skills using other methods and/or measurement instruments (written exams, oral exams, group work, etc.), which in turn are based on the evaluation criteria of regional regulations.

Standardized achievement tests are used to provide objective, reliable, and valid measures with greater use in the field of educational evaluation on a large scale. However, although school grades provide less evidence of reliability than standardized measures of academic achievement, they provide the most valid indication of a student's current level of achievement within a classroom environment, given that they are the closest assessment to the students' actual instruction (McCoach and Siegle, 2011). Using school grades to assess academic achievement also poses several problems, such as a lack of inter-rater reliability or comparability across teachers or schools (Marzano, 2000). The Spanish legal codes on curriculum and specific evaluation criteria do not resolve this question; however, this legal issue could be addressed.

Notably, knowledge of the differential characteristics of underachieving students is the basis to reverse underachievement (Renzulli and Reis, 1997; Chan, 1999, 2005). In the USA and to a lesser extent in China, studies have determined the factors that differentiate underachieving and normal-achieving students, both on the capacity continuum and for higher levels of cognitive ability (Baker et al., 1998; McCoach and Siegle, 2003b; Colangelo et al., 2004).

The scientific literature indicates that the primary reasons for underachievement in the majority of cases are (a) emotional problems (Siegle and McCoach, 2005); (b) difficulties in adapting to school or to the family context (Baker et al., 1998; McCoach and Siegle, 2003a); and (c) personal characteristics, such as low motivation, low self-regulation, or low self-concept (Reis and McCoach, 2000; Peixoto and Almeida, 2010; Dunlosky and Rawson, 2012).

Therefore, the differences between high ability students with high achievement and those with low achievement are explained by personal factors related to the use of self-regulation strategies, learning strategies and study techniques (McCelland et al., 1991; Colangelo et al., 2004).

A minor motivation in underachieving students is reflected in studies implemented both in the USA and China (McCelland et al., 1991; Schick and Phillipson, 2009; Hodis et al., 2011; Dunlosky and Rawson, 2012).

McCoach and Siegle (2003b) found that high ability students with high achievement and those with low achievement differ in their school attitudes and attitudes toward teachers, motivation, self-regulation, and valuation objectives. The last three factors contributed more in explaining the achievement differences between the two groups.

Results are inconsistent with respect to self-concept of underachieving students (Preckel and Brunner, 2015). Several studies report lower academic self-concept in underachieving students compared with non-underachieving students (Rimm, 2003), and gifted underachieving students have a lower general self-concept than non-underachieving students but not a lower academic self-concept (McCoach and Siegle, 2003a,b).

Baker et al. (1998) tested models related to personal, familiar, and scholar factors that explain underachievement in American adolescent students, finding that the model with three factors had a higher explanatory factor. Individual factors related to planning and the use of self-regulation strategies, self-perception of own abilities, and the quality of teacher-student relations were the variables that contributed more to explaining the differences between students with higher and lower achievement.

Therefore, although some of these factors have been identified, systematic studies in the Spanish cultural context are lacking. There are very few works on underachievement despite high failure and dropout rates. In Spain, the percentage of school failure or dropout during 2012–2014 was 23.5% (Eurostat, 2014), which is double the percentage for the European Union (11.9% for the same period). This considerable percentage of students experiencing school failure could be related to underachievement. Estimations of the percentage of underachieving students can vary depending on the socio-cultural context of the students involved. For example, in the USA, Rimm (1987) estimated that 50% of students have low achievement and high potential in Elementary Secondary Education, whereas Colangelo et al. (2004) made a lower estimate of 10% in a sample of high school students. In China, Phillipson (2008) calculated an empirical percentage of underachieving students that moved from 10% in the 50–59 capacity percentile bands (measured with a frequency distribution of the difference between ability and potential) to 32% in the higher 95 percentile bands in Primary Education. In Secondary Education, the percentage of underachieving students reached 53% in those whose capacity was in the higher bands.

Based on the above information and because most of the research on underachievement have been made in gifted underachievers and has not included overachieving students, the objectives of the present work were (1) to identify underachieving, normal-achieving, and overachieving students in Compulsory Secondary Education, (2) to identify the differential characteristics in each group of students based on personal factors, and (3) to analyze the educational implications of these characteristics in each group of students.

## Method

### Ethics statement

This study was carried out in accordance with the recommendations of Ethics Committee of Alicante University with written informed consent from all subjects.

### Participants

The cluster sampling technique was used with the school as the sampling unit. A total of 8 schools in the province of Alicante were included. A total of 1456 students in the first and second years of Compulsory Secondary Education (Educación Secundaria Obligatoria—E.S.O.) participated in the study. Of these, 56 were excluded due to coding errors or a lack of qualifications because they had special education needs or because they did not have parental consent, resulting in a total of 1400 students (*n* = 1400). A total of 53% of the students were male (47% female) with an average age of 12.5 years with a standard deviation of 0.67. A total of 52.4% of the students were from the first grade of E.S.O., and 47.6% were from the second grade of E.S.O. Due to the racial and ethnic homogeneity of the country, the majority of children were Caucasian (98%). Childhood socioeconomic status (SES) was indexed according to parental occupation. There was a wide range of socioeconomic status with a predominance of middle class children. This classification was based on the level of incomes and the level of studies of the families. The regional education counselors determined the childhood socioeconomic statuses (SES) through a questionnaire registered with the responses of the students. The variables used were parents' professions, professional situation, and level of studies, number of books at home, cultural and sporting activities and availability of technological means at home. The Chi-square test was used to determine differences between the gender of the sample (51.2% boys and 48.8% girls) and the gender of the national student population (51.3% boys and 48.7% girls), supporting the absence of gender differences between the sample and population (χ^2^ = 0.29, *df* = 1, *p* > 0.05).

### Measures

Measures of intelligence, learning strategies, goal orientations, and self-concept were collected during the academic year.

To measure intellectual ability, two tests were used: a general factor test and an aptitude test. Scale 2 of the Factor G test by Cattell (1994) and adapted into Spanish by TEA Ediciones was used to measure general and abstract intelligence. This scale produces an intelligence quotient (IQ) that measures general fluid intelligence. The reliability, obtained using the two-halves method and corrected with the Spearman-Brown formula, was 0.78 in first-year participants and 0.70 in second-year participants.

The other intelligences test used was the Battery of Differential and General Abilities (BADyG, Yuste et al., 2005). This Spanish battery measures the capacities and academic abilities of students. There are six subscales: Analogies (A), Series (S), Matrices (M), Completing sentences (C), Numerical Problems (P), and Figures Fit (E). Each subscale is measured with 32 items with five response options; only one option is correct, producing a total of 192 items. For this study, Cronbach's alpha values for each subscale were 0.83, 0.89, 0.79, 0.83, 0.77, and 0.87, respectively. Furthermore, a general intelligence quotient (IQ) could be obtained based on the punctuations from the distinct differential skills. The Cronbach's alpha of the total IQ was 0.83.

To measure learning strategies, we used the CEA *[Learning Strategies Questionnaire]* produced by Beltrán et al. (2006). The test evaluates four large strategies (Sensitization, Elaboration of information, Personalization, and Meta-cognitive strategies), from which only the last three were used in this study, as the sensitization scale refers to motivational and attitude-related aspects. The three scales employed for the evaluation of strategies include some subscales. The Elaboration scale is composed of the selection, organization, and information processing subscales. The Personalization scale includes the Creative and Critic Thinking subscale, the Recovering Information subscale and the Transference subscale. The Metacognition scale is composed of the Planning and Evaluation subscale and the Control and Monitoring Information subscale. To obtain the scores for these scales, students answered a total of 50 items indicating the extent to which each formulated strategy was true on a Likert scale from 1 to 5; we obtained sample Cronbach's alpha values between 0.87 and 0.71.

Goal orientation was measured through the CMA *[Academy Goal Questionnaire]* (García et al., 1998). This self-report instrument is a Spanish adaptation of the AGTD *[Achievement Goal Tendencies Questionnaire]* made by Hayamizu and Weiner (1991). The instrument contains 20 items and measures three types of goal orientations identified through factor analysis: learning goals, performance goals, and reinforcement goals. Students must answer on a Likert scale from 1 to 5 depending on the frequency of performance with each statement *(1* = *never; 5* = *always)*. The psychometric properties of the CMA have been analyzed with Spanish students at the primary, secondary, and university levels and have good levels of reliability and construct validity (González-Pienda et al., 2000; Navas et al., 2002). In our sample, the Cronbach's alpha values were 0.75 for learning goals 0.72 for reinforcement goals and 0.85 for performance goals.

To evaluate self-concept, we used the ESEA-2 *[Self-concept Evaluation Scale for Adolescents]* as expanded by González-Pienda et al. (2002). This questionnaire is a Spanish adaptation of the SDQ-II *[Self-Description Questionnaire]* by Marsh (1990a), which was validated in a study with 503 students in compulsory secondary education. The version employed in this study is composed of 70 items measuring 11 specific self-concept dimensions, which students must answer on a Likert scale from 1 to 6 depending on the extent to which they agree or disagree with each statement. The 11 specific dimensions are grouped in 4 general dimensions: academic self-concept, social self-concept, private/personal self-concept, and one general dimension of self-concept. In the authors' evaluation, all Cronbach's alpha values were between 0.73 and 0.91.

School grades were used as an indicator of academic achievement. Teachers provided full-term grades from nine subjects: Spanish language and literature, Natural sciences, Valencian/regional language, Social Sciences, Mathematics, English, Technology, Art Education, and Physical Education. The scores of the subjects of each course present a high reliability, with Cronbach's alpha values of 0.93 for the first course participants and 0.94 for the second course participants. In the present study, all of the subjects were compulsory for the students; thus, no choice of examination could affect the measurement of the latent construct (Korobko et al., 2008). A punctuation of academic achievement was calculated based on the factor scores obtained in a unique factor from the factor analysis of the grades in the nine subjects.

Once the viability of factor analysis was demonstrated (Barlett's χ^2^ = 9707.51, *df* = 36, *p* = 0.000; KMO = 0.95), an Exploratory Factor Analysis using maximum likelihood estimation identified a one-factor solution for the school grades that explained 69.57% of the variance; this indicates the unidimensionality of the model. All of the estimated factorial loadings remained over 0.78, with the exception of physical education, which was 0.66. Therefore, a single *factor score* was estimated using the regression method as a compound measure of current academic achievement.

### Procedure

Prior to data collection, the necessary permission was requested from the educational administration and school boards of the various schools. After obtaining these permits, the parents or legal guardians of the students had to provide the corresponding informed consent. Data collection was performed in the schools themselves during normal school hours. Data were collected by collaborating researchers previously trained in the standards and guidelines for data collection. Students and their parents participated voluntarily, and the parents signed an informed consent form that ensured data confidentiality at all times. The study was conducted from November to March over four sessions that each lasted an hour.

### Data analysis

First, a multiple regression analysis was performed to determine which of the two ability tests more accurately predicted academic achievement (Factor g test or BADyG). The regression analysis was made with the stepwise method, and the factor score was considered as a compound measure of current academic achievement as the criteria.

The multiple regression analysis used punctuations of IQ from the Factor G test and BADyG as predictors; the factor score obtained from the factor analysis made on the students' school grades in the nine subjects were taken as the criteria. The analysis showed that only the BADyG test significantly contributed to predicting the punctuation composed of academic achievement (*R* = 0.601, β = 0.60, *p* = 0.00), whereas the contribution of the Factor G test was not significant (β = 0.02, *p* = 0.43).

The regression method was employed to identify underachieving students along an all-capacity continuum. The regression method was calculated employing the IQ from BADyG as the predictor; the factor score was used as a compound measure of current academic achievement in the nine school grades as the criteria. The residual score or the difference between each individual's actual achievement score and his or her predicted achievement score were then examined. Three groups of students were formed using this method. Students with a residual punctuation higher than +1 were considered as overachieving, whereas students with a residual punctuation lower than −1 were classified as underachieving. The group with expected levels of achievement obtained punctuations between ±1.

To determine whether there were differences between the three groups, a one-way ANOVA was employed, followed by the *post-hoc* Games-Howell test, which is appropriate when there are groups with differing numbers of subjects and equal variances are not assumed.

## Results

The exploratory analysis of the data shows that all of the variables followed a normal distribution with values of skewness and kurtosis between +1/−1.

Table 1 shows the number of identified students in each subgroup with underachievement (1), normal achievement (2), and overachievement (3), according to the course and gender.

**Table 1:** **Descriptive statistics by gender, grade, and achievement group**.

	**Gender Frequencies**		**Grade/Year Frequencies**		**Total Freq**.	**Mean IQ**	**Mean Grades**
	**Boys**	**Girls**	**First**	**Second**			
Underachieving	137	81	127	91	218	101.78	4.30
Normal-achieving	532	437	496	473	969	100.29	6.30
Overachieving	70	143	111	102	213	101.11	8.39
Total	739	661	734	666	1400	100.00	6.30
	χ2 = 44.51^*^			χ2 = 3.57	*F* = 1.06	*F* = 541.0^*^	

* *p < 0.001*.

As can be observed in Table 1, the total numbers of underachieving, normal-achieving and overachieving students were 218, 969, and 213, respectively, which represent 15.6, 69.2, and 15.2% of the students. The percentages of first and second course students were similar (χ^2^ = 3.57, *df* = 2, *p* = 0.17), whereas the percentage of boys and girls significantly differed between each group (c^2^ = 44.51, *df* = 2, *p* = 0.00), showing a higher number of girls than boys in the overachieving group and a lower number of girls in the underachieving group. IQ punctuations were very similar for the three groups with no statistically significant differences (*F* = 1.06, *df* = 2, *p* = 0.64). However, there were significant differences between the academic qualifications with a higher mean in the overachieving group than the underachieving group (*F* = 541.0, *df* = 2, *p* = 0.00).

Table 2 shows the descriptive statistics of the measures in the learning strategies and goal orientations variables for each group. Students from group 3 (overachieving) obtained higher levels than students from groups 1 or 2 in all of the learning strategies variables, as well as goal orientations, whereas underachieving students received higher measures in performance goals and reinforcement goals.

**Table 2:** **Descriptive statistics of the punctuations obtained in learning strategies and goal orientations for each group**.

**Variable**	**Group**	***N***	***M***	***SD***
Selection	1	218	13.01	3.30
	2	969	13.66	3.22
	3	213	14.71	3.07
Organization	1	218	11.62	3.82
	2	969	13.09	3.83
	3	213	13.89	3.59
Elaboration	1	218	28.90	6.44
	2	969	30.78	6.33
	3	213	32.49	5.93
Elaboration scale	1	218	53.54	11.40
	2	969	57.53	11.24
	3	213	61.11	10.14
Creative and critical thinking	1	218	34.25	7.27
	2	969	35.52	7.22
	3	213	37.00	6.80
Recovering information	1	218	12.51	3.20
	2	969	13.31	3.22
	3	213	13.71	3.09
Transference	1	218	20.74	5.36
	2	969	22.26	5.35
	3	213	22.91	5.36
Personalization scale	1	218	67.52	13.59
	2	969	71.11	13.75
	3	213	73.62	13.03
Planning/evaluation	1	218	21.08	5.29
	2	969	22.47	5.32
	3	213	24.20	4.92
Regulation/monitoring	1	218	13.78	3.34
	2	969	14.78	3.24
	3	213	16.36	3.13
Meta-cognition scale	1	218	34.86	6.45
	2	969	37.25	6.38
	3	213	40.56	6.24
Learning goals	1	218	23.24	11.42
	2	969	25.46	11.23
	3	213	25.56	12.15
Achievement goals	1	218	25.68	7.40
	2	969	25.66	7.92
	3	213	24.93	9.15
Reinforcement goals	1	218	11.20	9.29
	2	969	11.15	9.18
	3	213	10.28	9.67

*Group 1, Underachieving students; Group 2, Normal achieving students; Group 3, Overachieving students; M, Mean; SD, Standard deviation*.

One-way ANOVA was performed to determine differences between the three groups in learning strategies and goal orientations (Table 3). There were statistically significant differences between the groups for all of the variables, with the exception of performance goals and reinforcement goals.

**Table 3:** **Results of ANOVA for learning strategies and goal orientations**.

**Variable**	**Source**	***SS***	***df***	***MS***	***F***	***p***	**Sense of differences^a^**
1	Between	324.25	2	162.13	15.70	0.00	3 > 2 > 1
	Within	14425.04	1397	10.32			
2	Between	590.73	2	295.36	20.50	0.00	3 > 2 > 1
	Within	20,124.08	1397	14.40			
3	Between	1388.99	2	694.50	17.56	0.00	3 > 2 > 1
	Within	55,249.69	1397	39.55			
4	Between	6167.84	2	3088.92	25.01	0.00	3 > 2 > 1
	Within	172,505.65	1397	123.48			
5	Between	814.33	2	407.17	7.93	0.00	3 > 2 > 1
	Within	71,763.48	1397	51.37			
6	Between	166.79	2	83.39	8.14	0.00	2 > 1, 3 > 1
	Within	14,318.23	1397	10.25			2 = 3
7	Between	565.89	2	282.95	9.86	0.00	2 > 1, 3 > 1
	Within	40,074.85	1397	28.68			2 = 3
8	Between	4113.81	2	2056.91	11.08	0.00	3 > 2 > 1
	Within	259,179.78	1397	185.53			
9	Between	1054.82	2	527.41	19.09	0.00	3 > 2 > 1
	Within	38,589.04	1397	27.62			
10	Between	737.69	2	368.85	35.02	0.00	3 > 2 > 1
	Within	14,712.68	1397	10.53			
11	Between	3548.31	2	1774.16	43.62	0.00	3 > 2 > 1
	Within	56,811.92	1397	40.66			
12	Between	1284.53	2	642.26	4.94	0.00	2 > 1, 3 > 1
	Within	181,784.99	1397	130.12			2 = 3
13	Between	98.00	2	49.00	0.76	0.47	1 = 2 = 3
	Within	90,454.42	1397	64.75			
14	Between	139.77	2	69.89	0.81	0.44	1 = 2 = 3
	Within	120,238.28	1397	86.07			

*Variables 1, Selection; 2, Organization; 3, Elaboration; 4, Information processing scale; 5, Creative and critic thinking; 6, Information recovering; 7, Transference; 8, Personalization Scale; 9, Planning/evaluation; 10, Regulation/Monitoring; 11, Meta-cognition Scale; 12, Learning goals; 13, Performance goals; 14, Reinforcement goals*.

a *p < 0.05*.

Games-Howell *post hoc* test for mean differences showed that group 3 (overachieving students) obtained higher measures than group 2, which had higher measures than group 1 (underachieving students) in all of the learning strategies variables with the exception of Information Recovering and Transference, for which no differences were detected between groups 3 and 2. However, overachieving students had higher punctuations than underachieving students.

On the goal orientation scales, the underachieving students had a significantly lower punctuation in learning goals than overachieving and normal-achieving students, whereas there were no differences between normal-achieving and overachieving students.

Therefore, the underachieving students showed significantly lower punctuations than the other two groups in all of the learning strategies variables and goal orientations variables. There were no significant differences for performance goals or reinforcement goals; however, underachieving students showed higher punctuations.

**Figure 1:**
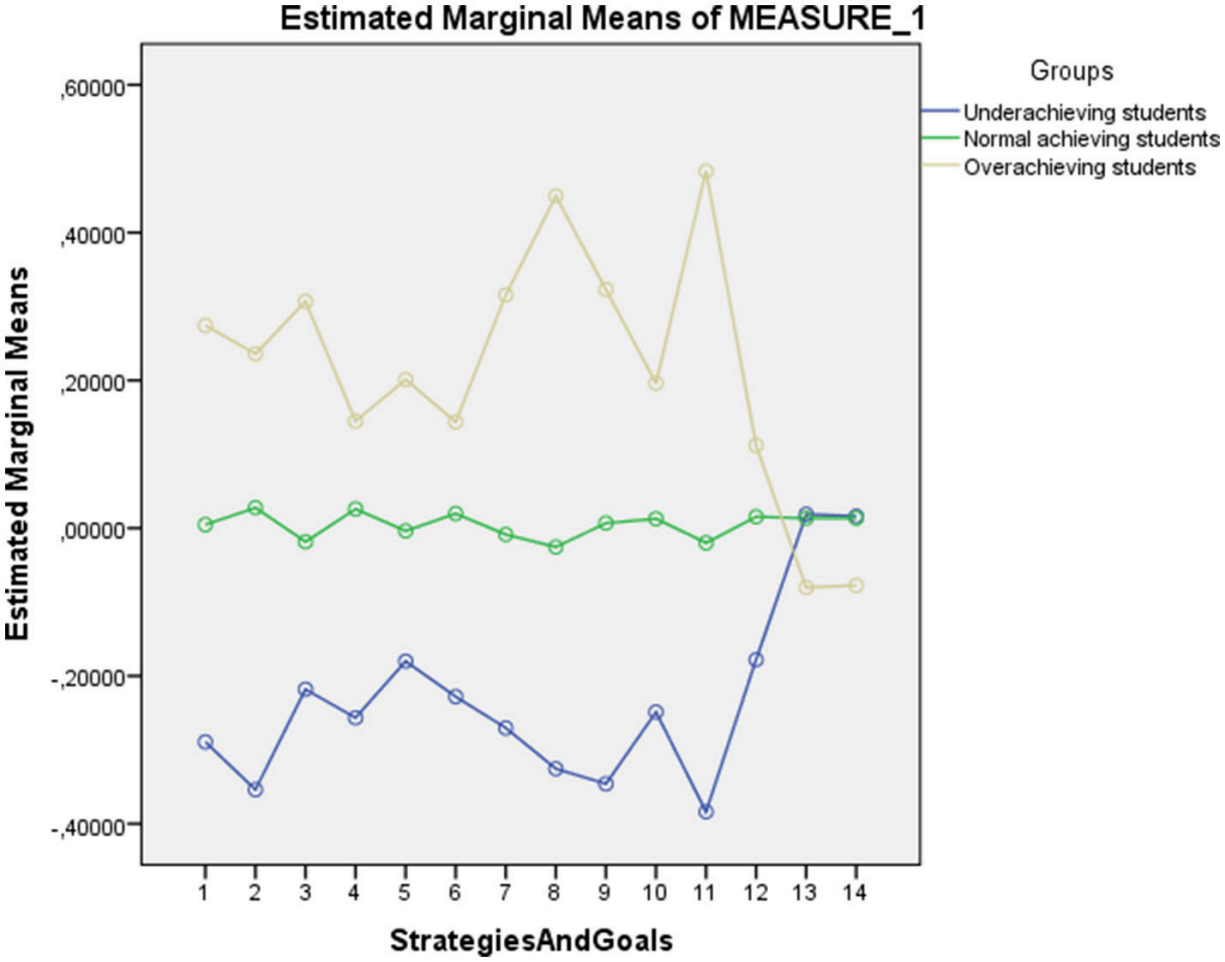
**Graphic representation of the standard scores in learning strategies and goal orientation measures for each group**.

The punctuations of the three groups are represented in Figure 1. To facilitate the comparison, all of the variables were converted into standard punctuations. The overachieving group had higher scores for all of the variables, with the exception of performance goals and reinforcement goals, for which they had lower punctuations, whereas the underachieving group had the opposite profile, with lower punctuations in all of the variables with the exception of performance goals and reinforcement goals, for which they had higher punctuations. The normal-achieving group had a flat profile with punctuations between groups 1 and 3 for all of the variables.

Tables 4, 5 show the results from the descriptive analysis and the mean differences of the self-concept variables, respectively. For many of the 15 self-concept evaluated factors in Table 4, students from group 1 had lower punctuations in the three second-order factors, especially in the factors related with academic self-concept, relation with parents, honesty, emotional stability, and private/personal general self-concept. In contrast, they received higher punctuations for physical capacity, relations with peers of the opposite gender and general social self-concept.

**Table 4:** **Descriptive statistics of the punctuations obtained in self-concept for each group**.

**Self-concept**	**Group**	***N***	***M*^a^**	***SD***
Mathematic	1	218	3.12	1.44
	2	969	3.61	1.45
	3	213	4.17	1.25
Verbal	1	218	3.22	1.07
	2	969	3.81	1.09
	3	213	4.45	1.00
Academic	1	218	3.50	1.12
	2	969	4.49	1.11
	3	213	5.31	0.71
Physical capacity	1	218	4.61	1.23
	2	969	4.51	1.23
	3	213	4.36	1.21
Physical appearance	1	218	4.06	1.29
	2	969	4.07	1.15
	3	213	4.25	1.07
Parent relationship	1	218	4.56	1.18
	2	969	4.94	0.99
	3	213	5.24	0.81
Honesty	1	218	4.04	1.05
	2	969	4.65	0.84
	3	213	4.99	0.73
Emotional stability	1	218	4.62	1.16
	2	969	4.87	0.97
	3	213	4.98	0.90
Peers relationship	1	218	4.89	1.01
	2	969	4.90	0.86
	3	213	4.92	0.83
Relation with peers of the same gender	1	218	5.01	0.74
	2	969	5.00	0.78
	3	213	5.06	0.72
Relation with peers of the opposite gender	1	218	4.11	1.05
	2	969	3.80	1.09
	3	213	3.54	1.09
General academic self-concept	1	218	3.28	0.90
	2	969	3.97	0.91
	3	213	4.74	0.73
General social self-concept	1	218	4.53	0.79
	2	969	4.45	0.72
	3	213	4.42	0.71
General private/personal self-concept	1	218	4.48	0.87
	2	969	4.82	0.83
	3	213	5.07	0.65
General self-concept measure	1	218	4.55	0.97
	2	969	4.89	0.84
	3	213	5.21	0.70

*Group 1, Underachieving students; Group 2, Normal-achieving students; Group 3, Overachieving students; M, Mean; SD, Standard deviation*.

a *Punctuations rescaled from 1 to 6*.

**Table 5:** **Results of ANOVA for self-concept variables**.

**Variable**	**Source**	***SS***	***df***	***MS***	***F***	***p***	**Sense of differences^a^**
1	Between	119.51	2	59.75	29.55	0.00	3 > 2 > 1
	Within	2824.80	1397	2.02			
2	Between	116.27	2	82.14	70.33	0.00	3 > 2 > 1
	Within	1631.47	1397	1.17			
3	Between	353.68	2	176.84	154.42	0.00	3 > 2 > 1
	Within	1599.75	1397	1.14			
4	Between	7.02	2	3.51	2.31	0.10	1 = 2 = 3
	Within	2123.73	1397	1.52			
5	Between	6.12	2	3.06	2.24	0.11	1 = 2 = 3
	Within	1908.74	1397	1.36			
6	Between	69.01	2	34.50	34.26	0.00	3 > 2 > 1
	Within	1406.80	1397	1.01			
7	Between	37.78	2	18.89	25.16	0.00	3 > 2 > 1
	Within	1049.04	1397	0.75			
8	Between	15.49	2	7.75	7.79	0.00	2 > 1, 3 > 1
	Within	1388.99	1397	0.99			2 = 3
9	Between	0.07	2	0.04	0.05	0.95	1 = 2 = 3
	Within	1104.91	1397	0.79			
10	Between	0.72	2	0.36	0.61	0.54	1 = 2 = 3
	Within	823.87	1397	0.59			
11	Between	35.44	2	17.72	14.92	0.00	1 > 2 > 3
	Within	1658.86	1397	1.18			
12	Between	200.51	2	100.25	126.95	0.00	3 > 2 > 1
	Within	1103.25	1397	0.79			
13	Between	1.45	2	0.73	1.34	0.26	1 = 2 = 3
	Within	754.83	1397	0.54			
14	Between	36.39	2	18.19	32.71	0.00	3 > 2 > 1
	Within	777.16	1397	0.55			
15	Between	46.86	2	23.43	32.93	0.00	3 > 2 > 1
	Within	993.86	1397	0.71			

*Variables 1, Mathematic self-concept; 2, Verbal; 3, Academic (other subjects); 4, Physical capacity; 5, Physical appearance; 6, Relation with parents; 7, Honesty; 8, Emotional stability; 9, Relation with peers; 10, Relation with peers of the same gender; 11, Relation with peers of the opposite gender; 12, General academic self-concept; 13, General social Self-concept; 14, General private Self-concept; 15, General Self-concept measure*.

a *p < 0.05*.

The one-way ANOVA results presented in Table 5 indicate that significant differences were found for most of the self-concept variables, with the exception of that related to physical capacity, physical appearance, relation with peers, relation with peers of the same gender, and social general self-concept.

The *post hoc* test indicated that underachieving students had lower punctuations than the other two groups in all of the cases in which significant differences were detected, with the exception of the punctuation obtained for relations with the opposite gender; this difference was higher than that for group 2, which in turn was higher than that in group 3. Overachieving students had lower punctuations in this self-concept aspect, whereas underachieving students had higher punctuations than the rest.

**Figure 2:**
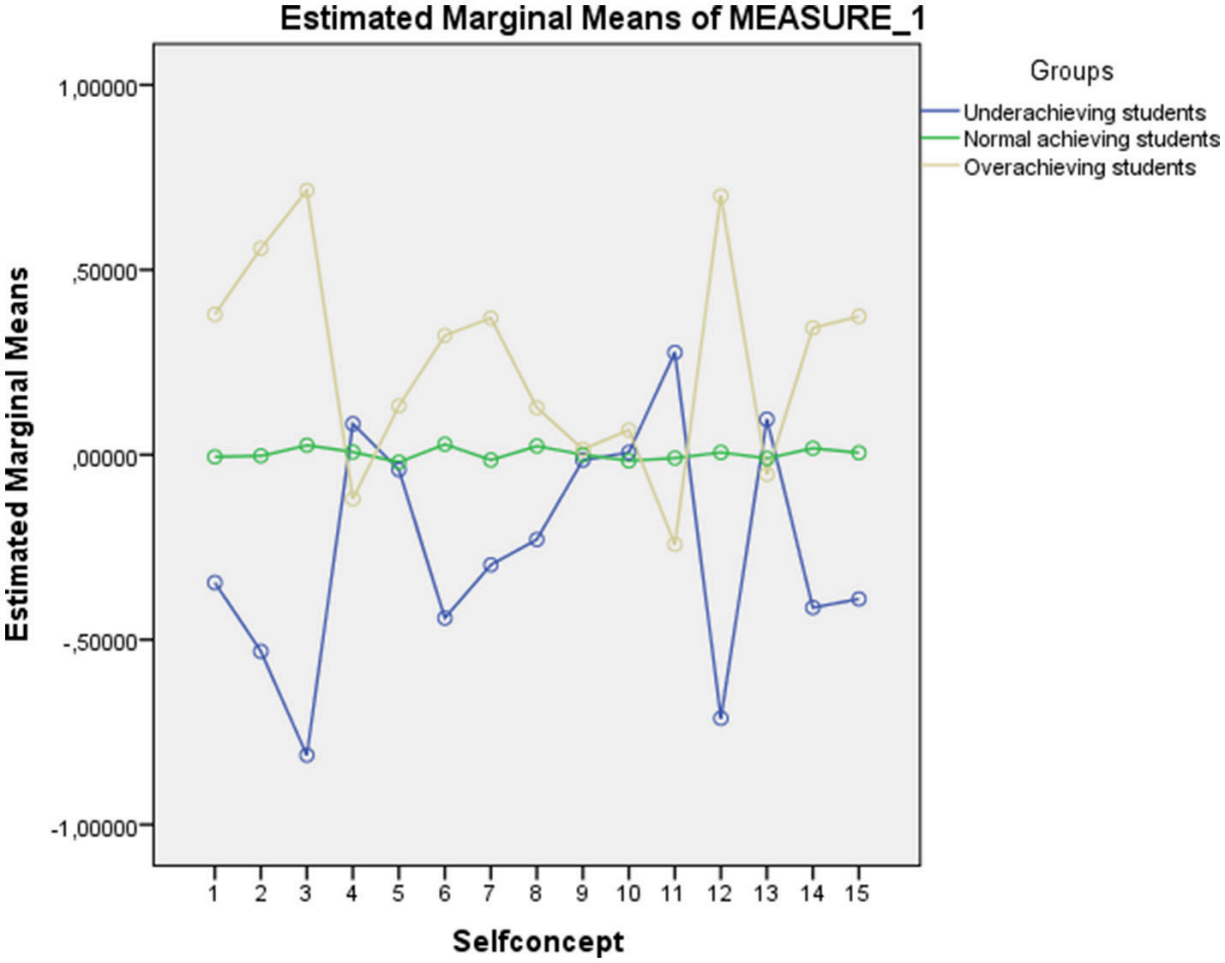
**Graphic representation of the standard scores obtained from the self-concept measures for each group**.

As shown in Figure 2, underachieving students' punctuations were below the majority of self-concept aspects, with the exception of physical capacity (4), relations with the opposite gender (11), and social general self-concept (13); however, these differences were only statistically significant for the relations with peers of the opposite gender. Notably, there was a flat profile in the normal-achieving group as well as higher punctuations in the overachieving group for most of the self-concept factors.

## Discussion

The regression method employed to identify underachieving students indicated a percentage of 16%, which is similar to rates in other studies (Lau and Chan, 2001; Colangelo et al., 2004; Phillipson, 2008); a nearly identical percentage was found for overachieving students. Although the regression method is considered the most adequate compared with other methods (such as the simple difference method, Lau and Chan, 2001; Phillipson, 2008), it tends to identify under- and overachieving students at a percentage near 16% when the standard residual value is fixed at 1 for the identification criteria (Ziegler et al., 2012). In general terms, the results from the different identification proceedings are not entirely coincident (McCoach and Siegle, 2011; Veas et al., 2016a). The results of this work indicate the construct validity of underachievement because the under- and overachieving groups showed defined characteristics according to theoretical expectations. They had nearly identical intellectual levels, demonstrated significantly distinct academic achievement, and showed significant differences in regard to most of the variables included in this study.

However, gender distribution differed between the groups, with a significantly higher number of boys than girls in the underachieving group and the opposite in the overachieving group. These results are similar to other studies reporting a higher risk for underachievement in boys (Reis and McCoach, 2000); however, some studies indicate no differences (Preckel and Brunner, 2015).

The results of this study also indicated significant differences between the under-, normal-, and overachieving students in most of the individual variables considered. In relation to learning strategies, underachieving students self-reported a lower use of all strategies, specifically, and generally considered, than the under and overachieving groups. When learning, underachieving students process less information and recover it with more difficulty; they also transfer or apply less of what they learn. When underachieving students plan, they evaluate and control the learning rhythm to a lesser extent. These results were found for a large sample of students that included the entire range of capacities; the results are similar to those obtained in studies with gifted underachieving students (Dowdall and Colangelo, 1982; McCoach and Siegle, 2003a; Colangelo et al., 2004) in which underachieving students show a systemic reduction in these strategies.

In contrast, overachieving students exhibited significant higher use of all of the learning strategies than underachieving and normal-achieving students. There were no differences for only the Information Recovering and Transfer strategies between normal and overachieving students. These results are interesting because they indicate that higher academic achievement in overachieving students is due to a major use of learning strategies; however, few studies have compared overachieving students with normal and underachieving students at all ranges of intellectual ability. Likewise, these results indicate that learning strategies represent a key variable in understanding the characteristics of under- and overachieving students and the role that these characteristics play for academic achievement. However, interventions to reverse underachievement should first focus on improving strategies before fostering motivational variables or academic self-concept (Preckel and Brunner, 2015).

The results of goal orientations as motivational variables indicate that the differences between under-, normal- and overachieving students are produced only for learning goals. Underachieving students showed minor punctuation compared with normal and overachieving students, whereas there were no differences in performance goals or social reinforcement goals. These results are similar to those obtained by Preckel and Brunner (2015), who only found positive relations for mastery goals and under- or overachieving students. Although there is a lack of studies comparing the goal orientations of under, normal- and over achieving students, findings regarding the general relationships between goal orientations and academic achievement are heterogeneous (Hulleman et al., 2010; Niepel et al., 2014).

With respect to self-concept, underachieving students showed lower punctuations than normal and overachieving students in all of the academic self-concept dimensions (mathematic, verbal, other areas, and in general academic self-concept). These results are primarily consistent with studies on academic self-concept in underachieving students (Rimm, 2003; Çakır, 2014; Preckel and Brunner, 2015). In the same manner, underachieving students showed lower scores than over and normal-achieving students in the dimensions of parent relationship, honesty and emotional stability; however, there was no difference between underachieving students and normal-achieving students for the last factor. All of these were specific dimensions of general personal self-concept, which also showed significantly lower punctuations; the same was noted for general self-concept. This minor private/personal general self-concept is in line with the results of McCoach and Siegle (2003a,b), which were obtained with the SAAS-R, an instrument for assessing characteristics in underachieving students, which was also employed in a study by Çakır (2014).

Underachieving students did not differ from the other groups in the self-concept dimensions related to parents' relationship, relation with peers of the same gender, or social general self-concept. However, they showed a higher self-concept with respect to relations with the opposite gender and physical capacity (not significant for the last component). Some studies showed that the relations perceived by the opposite gender do not have negative effects on achievement and that they appear to be mediated by the level of school engagement, which plays an important role in mediating these peer relationship effects, particularly for academic, and non-academic functioning (Liem and Martin, 2011).

In general, underachieving students' self-concept profile is lower than normal and overachieving students in all of the academic and personal dimensions, including relationship with parents. Underachieving students have a social self-concept that is similar to normal and overachieving students but higher in relations with the opposite gender and physical capacity. More studies are needed to determine if this self-concept in underachieving students is a result of compensation due to the lower academic and personal self-concept (Marsh, 1990a).

In summary, underachieving students appear to employ all of the learning strategies considered but to a lesser extent than normal and overachieving students. They also have fewer learning goals available and lower academic and personal self-concept. In contrast, overachieving students excel compared with under- and normal-achieving students in all of the above factors. If the three groups have similar intellectual levels, these differences in academic achievement appear to be associated with differential characteristics in learning strategies, goals, and academic and personal self-concept. The fact that overachieving students had superior performance compared with normal-achieving students appears to support this conclusion.

Therefore, in agreement with other studies (Gallagher, 1991; Emerick, 1992; Baum et al., 1995), any educational intervention focused on reverting the minor academic achievement in underachieving students must lead to simultaneously encouraging learning strategies, developing learning goals, and favoring the distinct general academic and personal self-concept dimensions. Self-regulating models in which goals, strategies and self-concept are integrated and have mutual relations and effects on academic achievement (Marsh, 1990b; Garcia and Pintrich, 1994; Pintrich, 2000; Zimmerman and Moylan, 2009; Valle et al., 2015a,b) appear adequate to guide educational interventions to reverse underachievement.

These interventions should follow certain criteria: to implement simultaneous changes in all of the factors in which underachieving students are at a lower level, to consider the main goal of the learning strategies; to focus on both enhancing specific self-efficacy and the general academic and personal self-concept, to positively influence academic achievement, and to be durable to produce the desired effects.

Other social and familiar variables are also important beyond those treated in this work. This is a limitation to our work that should be considered in future research.

Additionally, it is important to highlight the possibility to calibrate the school grades of students as the main measure of underachievement in Spanish schools (Veas et al., 2016b).

The inter-subject comparability approach is an appropriate model in which the influence of the difficulty level of the subjects and the proficiency level of the students can be adjusted according to Rasch's parameters. This approach has been tested (with some variation in the procedures) in various countries with positive results (Tasmanian Qualification Authority, 2006, 2007; Coe, 2007, 2008; Korobko et al., 2008).

However, it would also be necessary to determine whether these differences between groups are maintained when using other identification methods, such as the Rasch model, given that the percentage of underachieving students identified in a Spanish sample with the Rasch method was not the same compared with the simple difference method and the regression method (Veas et al., 2016a).

## Author contributions

JC Quantitative methods. Analysis of the sample. Reliability of the instruments. RG Theoretical review of the topic. Review of the references.

## Funding

The present work was supported by the Spanish Ministry of Economy and competitiveness (Award number: EDU2012-32156) and the Vice Chancellor for Research of the University of Alicante (Award number: GRE11-15). The third author is funded by the Spanish Ministry of Economy and Competitiveness (Reference of the grant: BES-2013-064331).

### Conflict of interest statement

The authors declare that the research was conducted in the absence of any commercial or financial relationships that could be construed as a potential conflict of interest.

## Footnotes

^1^Ley Orgánica 8/2013, de 9 de diciembre, para la Mejora de la Calidad Educativa [Organic Law 8/2013, from 9 December, for the Improvement of Education Quality]. *Boletín Oficial del Estado (España)*, 10 de diciembre de 2013 (10 December 2013), 295, 97858–97921.
